# Experimenter Effects on Pain Reporting in Women Vary across the Menstrual Cycle

**DOI:** 10.1155/2015/520719

**Published:** 2015-03-29

**Authors:** Jacob M. Vigil, Jared DiDomenico, Chance Strenth, Patrick Coulombe, Eric Kruger, Andrea A. Mueller, Diego Guevara Beltran, Ian Adams

**Affiliations:** Department of Psychology, University of New Mexico, 1 University of New Mexico, MSC03 2220, Albuquerque, NM 87131-1161, USA

## Abstract

*Background*. Separate lines of research have shown that menstrual cycling and contextual factors such as the gender of research personnel influence experimental pain reporting. *Objectives*. This study examines how brief, procedural interactions with female and male experimenters can affect experimentally reported pain (cold pressor task, CPT) across the menstrual cycle. *Methods*. Based on the menstrual calendars 94 naturally cycling women and 38 women using hormonal contraceptives (*M*_age_ = 19.83,  SD = 3.09) were assigned to low and high fertility groups. This assignment was based on estimates of their probability of conception given their current cycle day. Experimenters (12 males, 7 females) engaged in minimal procedural interactions with participants before the CPT was performed in solitude. *Results*. Naturally cycling women in the high fertility group showed significantly higher pain tolerance (81 sec, *d* = .79) following interactions with a male but not a female experimenter. Differences were not found for women in the low fertility or contraceptive groups. *Discussion*. The findings illustrate that menstrual functioning moderates the effect that experimenter gender has on pain reporting in women. *Conclusion*. These findings have implications for standardizing pain measurement protocols and understanding how basic biopsychosocial mechanisms (e.g., person-perception systems) can modulate pain experiences.

## 1. Introduction

It is generally accepted that gonadal sex hormones contribute to greater clinical and experimental pain experiences in women as compared to men [1–5]. Research thus far has demonstrated mixed findings on how the stage of menstrual cycle (and accompanying changes in steroids such as estradiol and progesterone) covaries with experimental pain sensitivity (e.g., [6–8]). Several studies have shown regular, isochronal fluctuations in pain sensitivity [9–13], while others have not found this effect [14–16]. Clearly, methodological factors (e.g., characteristics of samples, menstrual phase nomenclature, and nature of noxious stimuli) have contributed to these discrepancies [7, 17]. In our lab, we have also identified an important subject-level factor that appears to moderate changes in pain sensitivity across the menstrual cycle, namely, women's pair-bond status, qualified by the existence of a current romantic partner [18]. In two studies we found that only naturally cycling, pair-bonded women showed a positive correlation between the probability of conception across the menstrual cycle (based on their current cycle day) and experimental pain sensitivity (cold pressor task and ischemic pain). These associations were not found for single women or women using hormonal contraceptives [18]. These studies demonstrated that social psychological experiences may play an integral role in fertility-dependent fluctuations in extrinsic pain sensitivity.

Implicit and often unavoidable social experiences occur in laboratory and clinical settings in ways that can modulate the heuristical expression of verbal and nonverbal pain behaviors (see [19]). Several experiments have shown that female research participants tend to demonstrate heightened exogenous pain sensitivity following and during interactions with female experimenters and fellow female peers ([5, 20–22]; see also [23]). In contrast, there are mixed findings on the influence of exposure to males on women's pain sensitivity, as various studies show that the real or simulated presence of a man attenuates pain sensitivity in females [20, 24], while other studies have failed to show this effect [25–27]. One potential confounding factor that could explain these mixed findings is the possible role of women's fertility state during experimental participation. This raises the possibility that the gender of experimenters can affect momentary pain reporting of women differently across the menstrual cycle. Numerous domains of psychological functioning have been shown to vary isochronally across the menstrual cycle, including mate-perception processing, relationship satisfaction, risk avoidance, and social decision-making [28–35]. Most of this work did not consider how the researcher's gender may have influenced their results.

In the current study, we explore the hypothesis that experimenter gender influences experimental pain sensitivity differently for women depending on whether they are naturally cycling (i.e., versus hormonal contraceptive users) and whether they are at a relatively low or high fertility phase of their menstrual cycles. In theory, female-male interactions (and to a lesser extent female-female interactions) have the highest relevance to biological fitness and are thus the most important during peak fertility stages of the menstrual cycle. We therefore anticipated that the gender of experimenters who provide basic procedural instructions for conducting an experimental discomfort task (CPT) will have latent influences on pain reporting, particularly among women at high fertility phases of their cycles as compared to women at low fertility phases or women using a hormonal contraceptive. We estimated fertility state based on the probability of conception according to the participants' current cycle day (reverse counting method), and we simulated minimal procedural interactions with research personnel by limiting participant-experimenter interactions to only a brief consenting process and scripted experimental instructions. No researchers were present during the actual pain task. The experimental paradigm therefore allowed us to measure whether minimal social interactions with either a male or female researcher has latent influences on experimental pain reporting in healthy young women depending on the fertility state.

## 2. Methods

### 2.1. Participants

The study protocol was approved by the University of New Mexico's Institutional Review Board, and informed written consent was obtained from all participants. Undergraduate students received extra credit for an introductory psychology course for their participation. Participants who self-identified contraindications to the CPT were excluded from the study; these included taking any pain medications or having a problem that would increase risk of the CPT, including illnesses related to a cardiovascular disorder (e.g., high blood pressure, heart problems, or heart rhythm concerns), history of fainting or seizures, history of frostbite, having an open cut, sore or bone fracture on the limb to be immersed in water, or a history of Reynaud's phenomenon. All participants signed up via the University of New Mexico Sona website for psychological research volunteers. To be included in the analysis, females had to be naturally cycling (i.e., nonhormonal contraceptive use) or using a hormonal contraceptive and had to have not yet experienced menopause. The final sample consisted of 132 women with complete data for inclusion in the study (18–48 yrs, *M*_age_ = 19.83, SD = 3.09; 39.4% European American, 40.2% Latin American, 20.4% other/not specified ethnicity; 71.2% naturally cycling, 28.8% hormonal contraceptive users).

### 2.2. Procedures

Experimenters (7 females and 12 males) assisted participants through the protocol. The gender of the researcher was operationalized as the outward expression or appearance of culturally defined masculinity and femininity. Forty-seven percent of participants (*n* = 62) were processed by a female experimenter, and fifty three percent of participants (*n* = 70) were processed by a male. The proportion of female/male and Hispanic/non-Hispanic White researchers did not differ for women in the low versus high fertility group (*p*s > .10).

Experimenters followed a scripted protocol in order to minimize any possible influences from interpersonal factors (e.g., duration of conversations, eye contact, and personal factors) that are not contributing to researcher's gender identity. After participants were finished with the consenting process, they were measured for physical characteristics (e.g., height, not used in the current study), which requires minimal and noninvasive physical contact between the subject and the experimenter, and subjects provided information about their menstrual functioning, which usually took between 3 to 5 minutes to complete. Afterwards, participants were guided to an assessment room where they were left alone to fill out a demographic questionnaire and watch a short video regarding the instructions for the CPT. The video depicted a male researcher performing the experiment with explicit directions provided in a female voice. The video also described the directions for using the cold pressor apparatus and how to indicate the various pain measurements. Again, to minimize exposure to the participant, the experimenter was not present while the participants completed the demographic survey and video portion of the experiment; this portion of the experiment usually lasted between 20 and 30 minutes.

Next, the participants were escorted to the room containing the cold pressor apparatus and a small laptop with a user interface pain assessment program. This program recorded the development of pain as participants indicated (by pressing a corresponding icon) when the discomfort sensation began to feel painful (pain threshold) and when they quit the task (pain tolerance). Finally, the researcher addressed any questions the participants had about CPT instructions, and the participants were told that they could choose when to begin the task. The experimenter then left the room and closed the door behind her/him. The participant performed the CPT without the presence of the researcher in order to minimize the possible influence of audience effects. Researchers monitored the participants via a hidden video camera during the task to ensure the adherence to instructions. None of the resulting CPT sessions were interrupted mid experiment by an experimenter. In total, the participants interacted with the researchers for roughly 5–10 minutes (e.g., for the consent process, to take body measurements, to escort participants to the various laboratory rooms, and for answering questions) throughout the experiment up to the CPT.

#### 2.2.1. Questionnaires

A basic questionnaire created by our lab gathered information about sex, age, ethnicity, education, and family background. The menstrual-related information included whether or not the participant was currently menstruating, usage and type of hormonal contraceptives, average number of days in their typical menstrual cycle, and number of days since their last menstrual cycle (from the date of assessment). Participants were provided a calendar to calculate their responses.

### 2.3. Cold Pressor Task

#### 2.3.1. Cold Pressor Apparatus

Participants were seated in a chair between the cold pressor apparatus (on the participant's left side) and the laptop computer (right side) in a small room (2.0 m × 2.5 m). The mechanical CPT device was an Isotemp 6200R28 refrigerated bath circulator (reservoir size: 29 cm × 16.5 cm × 22.4 cm). The machine circulates the water automatically and maintains a consistent water temperature by dual heating and cooling actions. The water temperature was set to 5°C (known to produce a range of pain tolerance levels with only minimal ceiling effects [36]). Small differences in water temperature (2°C) can have significant effects on pain sensitivity measures [37], and all the participants in the current study experienced water temperatures within 0.5°C of each other.

#### 2.3.2. Cold Pressor Procedures

The pain assessment program displayed an initial screen with the general CPT instructions. The researcher verbally reiterated the instructions by describing that when participants choose to both begin (after the researcher left the room) and end the task (at maximum pain tolerance) they were to perform two simultaneous actions. To begin the task (and initiate the pain assessment program), participants were instructed to first indicate their baseline (premanipulation) pain severity along a standard visual analog scale (VAS, 0–10 from* no pain* to* worst pain imaginable*), while simultaneously submerging their left hand into the cold water to a marked line on the wrist (2.5 cm above the wrist joint). To end the task, participants were instructed to indicate this preference by clicking on a corresponding icon on the computer screen while simultaneously lifting their hand out of the cold pressor apparatus. Participants were also instructed to immediately indicate their pain threshold and total pain tolerance times by clicking on the corresponding buttons on the computer screen.

The CPT was observed on a video monitor from a remote location, and the researcher returned to the experimental room to debrief the participant once they retracted their hand from the water or after the maximum duration of 5 minutes had occurred (the participants were not informed of this time limit before beginning the pain task). Following debriefing, participants were asked to rest for five minutes to ensure they no longer felt any physical discomfort from involvement in the study and that their heart rate had returned to resting.

### 2.4. Data Analyses

Individual-level fertility level was calculated using the Wilcox findings which provide a precise estimate of the probability of conception based on a standardized 28-day cycle [38]. These estimates calculated the probability of conception relative to intercourse on a given cycle day (counting from onset of previous menses). This variable was then split into two groups, low versus higher fertility, according to the mean value among naturally cycling women, similar to previous studies [39].

The pain scores included the participant's pain threshold and pain tolerance (measured in seconds after submersion). Lower threshold and tolerance scores are interpreted as indicating greater CPT pain sensitivity as is common in the pain literature. Multilevel (random-effects) models were used to examine the separate effects of experimenter's gender (coded 0 = male, 1 = female) and fertility stage of the participant's menstrual cycle (coded 0 = not fertile, 1 = fertile) on the pain scores separately in the naturally cycling and hormonal contraceptive-using women; age, baseline pain intensity (prior to CPT), weight, and experimenter's ethnicity (coded 0 = White, 1 = Hispanic) were entered as covariates. Multilevel models were preferred over linear regression or analysis of covariance because the same 19 experimenters were used across all 132 pain tasks; our multilevel models account for the repeated measures across experimenters. We also reported Cohen's *d* (mean difference/mean standard deviation [40]) to provide an estimated effect size for the group comparisons, even though it does not control for the covariates or account for repeated measures among the personnel.

All of our analyses were conducted in R v3.0.2 [41], using restricted maximum likelihood estimation in the package* lme4* v1.1-7 [42].

## 3. Results

In order to examine whether gender of the experimenter interacts with fertility stage to influence pain reports, a multilevel model was estimated for each of the pain scores (pain threshold and pain tolerance) separately for natural cycling and hormonal contraceptive-using women. For these models, experimenter gender, fertility stage, and the gender × fertility stage interaction terms were entered as predictor variables along with the covariates (age, baseline pain, weight, and experimenter ethnicity). For women on a natural cycle, a significant experimenter gender × fertility stage interaction term emerged for pain tolerance, *B* = −99.30, *z* = −2.21, and *p* = .027, and for pain threshold, *B* = −20.48, *z* = −1.97, and *p* = .049; the interaction term was not significant for women using hormonal contraceptives (*p*s > .70).

**Figure 1:**
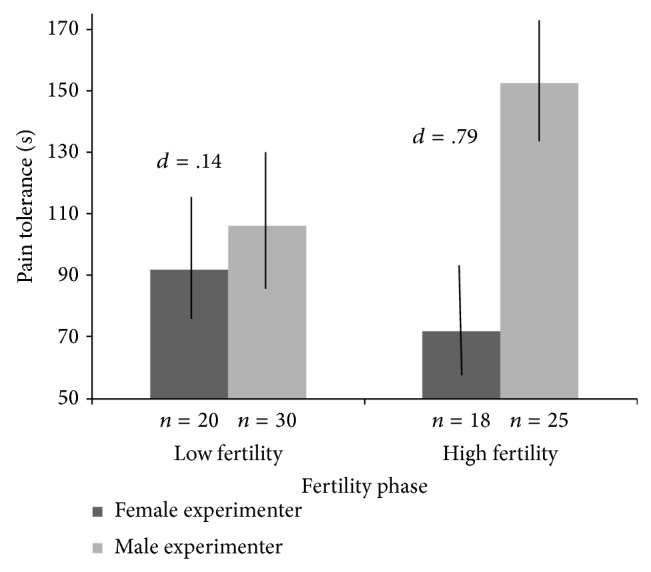
Plot of significant fertility × experimenter gender interaction for pain tolerance in naturally cycling women. Higher values on the *y*-axis indicate lower pain sensitivity. Fertility phases are represented by values lower than and greater than the mean probability of conception based on the women's current cycle day. Bars indicate standard errors of the mean.

Follow-up analyses examining the effect of experimenter gender on pain tolerance reports separately for naturally cycling and hormonal contraceptive-using women at the high and low fertility stages (entering the covariates) showed a significant effect of experimenter gender for the naturally cycling women at high fertility only, *B* = −114.36, *z* = −2.51, and *p* = .012. The effect of gender was not significant either for natural cyclers at low fertility (*p* = .298) or for women using hormonal contraceptives in either of the fertility groups (*p*s > .10). These effects are illustrated in Figure 1, which shows the bivariate relations (not controlling for covariates) between the experimenter's gender and pain tolerance for naturally cycling women at high and low fertility phases of their menstrual cycle (the results are the same when the covariates are controlled). As shown in Figure 1, women who were at high fertility and interacted with a male experimenter prior to the CPT reported higher pain tolerance (81 secs) than women who were at high fertility and interacted with a female experimenter (*d* = .79).

Follow-up analyses examining the effect of experimenter gender on pain threshold separately for naturally cycling and hormonal contraceptive-using women at the high and low fertility stages (entering the covariates) failed to show any effect of experimenter gender for naturally cycling women at high fertility, *B* = −14.38, *z* = −1.57, *p* = .116, and *d* = .40. The effect of gender was again not significant either for natural cyclers at low fertility (*p* = .914) or for women using hormonal contraceptives in either of the fertility groups (*p*s > .90).

## 4. Discussion

This study shows for the first time that the gender of persons in the immediate social context influences how a female research participant subsequently reports pain experiences differently across fertility phases of the menstrual cycle at the time of participation. At a fertile phase of their cycle, naturally cycling women showed significantly higher pain tolerance following interactions with a male, but not a female, experimenter. These differences were not found for women at a relative infertile phase or if they were using hormonal contraceptives. In general, these findings build on previous research on social relationships and menstrual-dependent changes in pain sensitivity [18] by showing that minimal procedural interactions with laboratory personnel of different genders can influence momentary pain reporting based on a probability of conception (i.e., fertility).

These results are consistent with the broader literature showing that females have evolved numerous adaptations for experiencing fertility-dependent changes in basic components of social cognition (e.g., feelings, dispositions, and judgments) in ways that could modify affiliation and avoidance with others, particularly men. Menstrual-related changes in social cognition have been found across several domains of psychological functioning, including romantic relationship preferences, relationship satisfaction, risk avoidance, and social decision-making [25, 28–35, 43]. Along the same lines, a social-signaling perspective of pain predicts that human-suffering behaviors heuristically operate at an expressive level for selectively advertising capacity/fitness cues (via pain concealment) or trustworthiness/vulnerability cues (via pain expression) to different affiliates [19, 44–47]. It is within reason, therefore, to predict that exposure to and interactions with unrelated males have more biological relevance to women when they are most fertile. Given this, our findings are consistent with two general, though antithetical, explanations.

The first explanation for reduced pain sensitivity around ovulation in the presence of a male is that reduced pain sensitivity facilitates sexual interaction and successful insemination by reducing pain intensity associated with the copulation act itself. Previous research has shown that at least one-third to half of females experience pain during their first sexual encounter [48–50]. Women also experience more sexual dysfunctions related to pain than males, and there are several disorders that cause dyspareunia (i.e., painful sexual intercourse; e.g., vestibulodynia, interstitial cystitis/painful bladder syndrome, pelvic floor hypertonus, and vaginismus), which has been estimated to affect as many as 9%–15% of women [51, 52]. The fact that so many women experience pain during first penetration, if not for the remainder of their reproductive years, suggests that physical pain serves a chief role in the female's decision to engage in sexual activity. The dampening of pain during ovulation might function as a “transitionary mechanism” that enables the transition from painful sex to pleasurable sex, thereby preventing avoidance reinforcement and ultimately unsuccessful insemination and impregnation. Male audience-induced hypoalgesia with increased ovulation could also facilitate short-term reproductive strategies [53–55], for instance, by mitigating pain that may result from multiple sexual encounters in a short time frame during the window of ovulation to ensure impregnation. Based on sexual selection theory, this effect should vary according to numerous additional conditional factors of the woman (e.g., age, pair-bond status, and fitness markers) and contextual factors such as the attractiveness and perceived status/dominance of the male audience [56–61].

The second explanation for reduced pain sensitivity around ovulation in the presence of a male is that decreased pain sensitivity following exposure to unrelated males operates alongside other psychological and somatic changes across the menstrual cycle that evolved to reduce the risks of rape, ultimately facilitating rape avoidance [62, 63]. Studies have shown that women perceive men as more sexually coercive [64] and engage in fewer risky behaviors when they are experiencing higher levels of fertility, despite demonstrating higher overall activity levels during this phase of the menstrual cycle [65–67]. In a unique study, researchers found that women in their most fertile phase showed an increase in handgrip strength but only following exposure to a sexual coercion scenario [68]. Another study has shown a similar pattern of increased handgrip and quadriceps strength and greater overall athletic performance (e.g., successful jumps in a fitness test) during midcycle in naturally cycling women [69, 70]. Taken together, these findings suggest that women have evolved capacities to facilitate rape avoidance that vary across the menstrual cycle. Should such an adaptation exist, changes in pain perceptions that function to avoid risk would also be moderated by numerous personal factors such as women's attractiveness, pair-bond status, and location of family residence [71]. Other psychological factors may also influence women's ability to avoid rape. For example, it was reported that women who perceive themselves as having better physical condition performed more rape avoidance behaviors [72]. Moreover, other researchers demonstrated that women reported greater race bias as a function of increased risk of conception [73] and that there is a negative correlation between experiences of disgust and age and a positive correlation between experiences of disgust and increased risk of conception [74]. Still, the hypothesis that changes in pain perceptions function to facilitate rape avoidance remains challenging given that heightened pain sensitivity seems to be a better promoter of threat aversion than the pattern observed in the current study (hypoalgesia). Further research is therefore needed to elucidate how changes in pain sensitivity may correspond to changes in sexual motivations and rape avoidance strategies across the menstrual cycle under varying environmental and situational contexts.

The proximate mechanisms that may process social experiences on pain perception across the menstrual cycle remain unclear. For example, one possibility is that women may experience elevations in oxytocin in the presence of unfamiliar males. It is well established that oxytocin is involved with social behaviors such as social distance and eye contact (see [75]). Specifically, intranasal oxytocin decreased the social distance with male experimenters [76]. Oxytocin also dampens pain sensitivity, and people with chronic pain conditions (e.g., fibromyalgia) may have lower basal levels of oxytocin than healthy people [77–79]. Estradiol also appears to interact with oxytocin and increases its analgesic effects [80, 81], which may account for lower levels of perimenstrual-related pain during high fertility phases of the menstrual cycle. Hence, basal oxytocin or possibly other hormones (e.g., testosterone) could be a proximate mechanism that can explain the association between fertility (which is associated, even if imperfectly, with estradiol [82]) and changes in pain sensations in normally ovulating women in the presence of and following exposure to unfamiliar males. Pain-specific brain activity has been shown to vary across the menstrual cycle [83, 84], and a better understanding of the role of social psychological processes may be important to contribute to this and similar lines of research.

The current findings may also have direct implications for health care providers and research investigators. Pain is often considered the fifth vital sign alongside temperature, heart rate, blood pressure, and respiratory rate, and pain measurement is central for effective and patient-centered care. Many implicit and currently unavoidable social cues, such as examiner characteristics, have been shown to contribute to measurement error in patient pain reporting [23]. The current investigation highlights the need to further understand unmeasured sources of reporting biases in clinical settings. These findings also highlight the methodological confounding factor of reporting pain across the menstrual cycle when experimenters are of different genders, which could have affected numerous previous investigations (see [13, 85]). Previous studies relied on pain reporting using paradigms that did not tightly control or account for experimenter characteristics and other contextual factors. Therefore, the possibility that women's menstrual functioning may interact with experimenters' characteristics to affect pain reporting should warrant caution for researchers assessing multiple lines of basic and applied research.

In addition to these implications, a discussion of our study's limitations is warranted. One limitation is the relatively small sample size, disallowing examination of additional social factors such as quality of peer and romantic relationships, which have been shown to covary with experimental pain reports in women [18, 19, 86]. Other general methodological limitations are that (a) the study did not control for handedness, which is known to influence CPT measurements [87]; (b) reactions to CPT might not predict reactions to other forms of painful and nonpainful extrinsic stimuli; (c) results from American university students might not generalize to different ages, cultures, and patient categories; and (d) self-reports of menstrual cycling using the counting-back method (based on current cycle day) might be biased and prone to error. It is also possible that the interaction that participants had with experimenters, which required experimenters to briefly touch the subjects (to facilitate body measurements), may have influenced the results. For example, some experimenters may have touched the female participants slightly differently, and the touching behaviors in and of themselves contributed to differences in pain tolerance. Future experimental studies using multiple methods (e.g., endocrine, neurological, and subjective reporting) that can circumvent the current study limitations (e.g., small sample size) are needed to better understand how menstrual-related changes in psychophysiological processes correspond to subjective pain experiences.

Nonetheless, the current findings have wide-reaching implications for (a) standardizing pain measurement protocols, (b) understanding basic biopsychosocial pain-related processes, (c) addressing clinical pain experiences in women, and (d) understanding how social interactions influence felt pain. Previous investigations delineated both significant and negligible associations between menstrual cycling and pain intensity, and the current study highlighted a significant methodological-level confounding factor, namely, the gender of laboratory personnel interacting with participants, which has not been controlled in most previous studies. Finally, the current results have implications for understanding how social experiences and the motivation to either affiliate or avoid selective social affiliates in the immediate context may induce changes in somatic (e.g., sensory and perceptual) functioning, including exogenous pain sensitivities.
